# Evaluation of the PRE-DELIRIC delirium prediction tool on a general ICU

**DOI:** 10.1186/cc14559

**Published:** 2015-03-16

**Authors:** J Hanison, S Umar, K Acharya, D Conway

**Affiliations:** 1 Manchester Royal Infirmary, Manchester, UK

## Introduction

Delirium is a frequently occurring complication of critical care, occurring in approximately 45% of unplanned UK ICU admissions [1]. The presence of delirium in critical care is an independent risk factor for mortality; for every day of delirium, there is an additional 10% relative risk of death at 1 year [2]. A delirium prediction tool PREDELIRIC has been recently developed and calibrated in a multinational project [3]. This study aimed to determine the utility of PRE-DELIRIC on our ICU.

## Methods

This study prospectively investigated 41 patients. Medical and surgical general ICU patients were included after 24 hours of sedation and mechanical ventilation. The researchers calculated PRE-DELIRIC scores for each patient. PRE-DELIRIC involves recording 10 variables, submitted into an online algorithm that estimates the percentage risk of delirium. We diagnosed delirium with the CAM-ICU which was performed 12 hourly [4].

## Results

**Figure 1:**
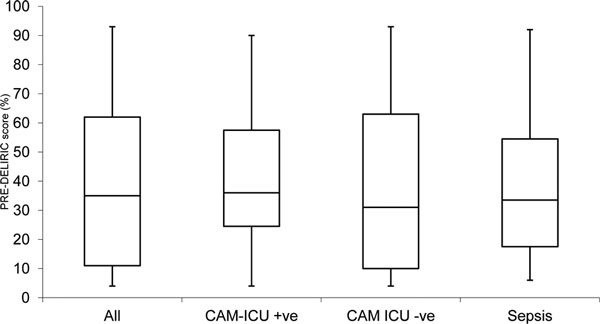
**Range of PRE-DELIRIC scores**.

The PRE-DELIRIC scores predicted a mean rate of delirium of 39%. PRE-DELIRIC risk scores ranged from 4 to 93% (Figure 1). Six (15%) patients developed delirium in the first 24 hours following extubation. Fifteen (37%) of patients were predicted 20% or less probability of delirium. Twelve (29%) patients developed delirium at any point during their ICU stay. This resulted in 36 total delirium bed-days.

## Conclusion

Our observation that <30% of patients experienced delirium is less than the reported prevalence in similar settings and our own audits. This study demonstrates that there is some agreement between recorded rates of delirium and predicted rates using PREDELIRIC. We suggest that PRE-DELIRIC can be used in quality/audit work on UK ICUs in order to assess attempts to improve the management of delirium. Further work is required to assess the utility of PRE-DELIRIC as a risk assessment tool in individual patients.
